# Diet-Induced Obesity Promotes Colon Tumor Development in Azoxymethane-Treated Mice

**DOI:** 10.1371/journal.pone.0060939

**Published:** 2013-04-03

**Authors:** Iina Tuominen, Leina Al-Rabadi, Dimitris Stavrakis, Iordanis Karagiannides, Charalabos Pothoulakis, James M. Bugni

**Affiliations:** Division of Digestive Diseases, David Geffen School of Medicine at University of California in Los Angeles, Los Angeles, California, United States of America; Rush University Medical Center, United States of America

## Abstract

Obesity is an important risk factor for colon cancer in humans, and numerous studies have shown that a high fat diet enhances colon cancer development. As both increased adiposity and high fat diet can promote tumorigenesis, we examined the effect of diet-induced obesity, without ongoing high fat diet, on colon tumor development. C57BL/6J male mice were fed regular chow or high fat diet for 8 weeks. Diets were either maintained or switched resulting in four experimental groups: regular chow (*R*), high fat diet (*H*), regular chow switched to high fat diet (*RH*), and high fat diet switched to regular chow (*HR*). Mice were then administered azoxymethane to induce colon tumors. Tumor incidence and multiplicity were dramatically smaller in the *R* group relative to all groups that received high fat diet at any point. The effect of obesity on colon tumors could not be explained by differences in aberrant crypt foci number. Moreover, diet did not alter colonic expression of pro-inflammatory cytokines tumor necrosis factor-α, interleukin-6, interleukin-1β, and interferon-γ, which were measured immediately after azoxymethane treatment. Crypt apoptosis and proliferation, which were measured at the same time, were increased in the *HR* relative to all other groups. Our results suggest that factors associated with obesity – independently of ongoing high fat diet and obesity – promote tumor development because *HR* group animals had significantly more tumors than *R* group, and these mice were fed the same regular chow throughout the entire carcinogenic period. Moreover, there was no difference in the number of aberrant crypt foci between these groups, and thus the effect of obesity appears to be on subsequent stages of tumor development when early preneoplastic lesions transition into adenomas.

## Introduction

Obesity is a worldwide epidemic and strongly predisposes to several diverse disease states including cancer. Obesity, as estimated by body mass index (BMI), is significantly correlated with increased risk of multiple cancers, including those of the esophagus, thyroid, colon, kidney, endometrium, and gall bladder [1]. Importantly, colon cancer risk increases dramatically with BMI, as each stepwise increase of 5 kg/m^2^ is associated with an 18% increase in cancer risk [2], and a comparable increase in adenoma risk [3].

Adipose tissue is an active metabolic organ and a source of hormones, cytokines, and growth factors. Obesity is considered a state of “low grade inflammation” due to its association with increased circulating levels of proinflammatory cytokines, associated with chronic inflammation, insulin resistance, and other aspects of metabolic syndrome [4]. The effect of adipose-derived growth factors and cytokines on colon cancer progression is not fully understood. Some studies have shown effects of leptin and adiponectin [5], [6], while others have not [7], [8]. Additionally, tumor necrosis factor α (TNF-α), which is elevated in obese states, could play a role [9]. Expansion of adipose tissue is associated with elevated triglyceride and low-density lipoprotein cholesterol levels, and hyperinsulinemia, which are potential mediators of tumor development [10], [11]. It is hence possible that circulating, distinct from adipose-derived, factors such as free fatty acids or insulin are also crucial in mediating the pro-tumorigenic effect of obesity and high fat diet (HFD). Thus the effects of diet-induced obesity (DIO) on colon cancer may result from a combination of direct effects of dietary components in the colonic epithelium, secreted factors from adipose tissue, or systemic changes related to type II diabetes.

Because obesity is associated with higher consumption of fats and dietary lipids that can affect colon cancer development independently of adiposity, it is difficult to separate these factors epidemiologically and empirically. Components of a HFD and the type of fatty acids consumed have potent effects on colon tumorigenesis, suggesting that they may directly influence cancer risk [12]. The mechanisms by which various dietary fats increase colon carcinogenesis independently of adiposity are not fully understood, but may involve effects on oncogene expression in the colonic mucosa [12], [13], secretion of bile acids [14], or oxidative stress from hyperoxidizable triglycerides [15].

Chemical carcinogenesis studies in murine models generally show a tumor enhancing effect of HFD feeding and associated obesity [14], [16]. A western-style diet high in fat and low in several key nutrients may increase tumor formation even without carcinogen treatment [17]. Moreover, genetic mouse models suggest that obesity that develops without HFD feeding leads to tumor promotion. For example, both leptin deficient (ob/ob) and leptin receptor deficient (db/db) mice show increased sensitivity to colon tumor development [7], [18]. These animals suffer from immune and hormone abnormalities that may confound the effect of excess adipose tissue on carcinogenesis. DIO models ideally mirror human obesity and metabolic syndrome. They typically, however, have the same confounding element than human studies – the possible independent effects of dietary fat on the colonic epithelial cells and metabolic functions.

We took a novel approach to determine the effects of obesity, without ongoing HFD, on colon tumor development. After inducing obesity with HFD, mice were administered a regular diet prior to treating with azoxymethane (AOM) to induce colon tumors. Therefore, during the entire carcinogenic period, these mice were on the same diet as control mice, and yet both incidence and multiplicity of colonic adenomas were significantly increased. This is the first study to show that obesity itself enhances colon tumor development, without the confounding effects of ongoing HFD or genetic disruption.

## Methods

### Feeding and azoxymethane (AOM) treatment

Five week old C57BL6/J mice were purchased from the Jackson Laboratory and were fed regular chow or HFD (PicoLab 5053 diet with 13% of calories from fat or Open Source Diet D12451 with 45% of calories from fat, Table 1) for 8 weeks, and then were either maintained on these diets or switched resulting in 4 experimental feeding groups: regular chow (*R*), HFD (*H*), regular chow switched to HFD (*RH*) and HFD switched to regular chow (*HR*). Two weeks after the dietary switch, mice were given 5 weekly injections of AOM to induce colon tumors. So that more obese animals did not receive a higher dose of the carcinogen, all animals were given the same amount of AOM that corresponded to 10 mg/kg per injection calculated for the average body weight of *R* group mice. For molecular analyses, mice were euthanized 24 hrs after the final AOM treatment (N = 5) together with age and diet matched animals that did not receive AOM (N = 2 or 3). For tumor studies, mice were euthanized 23 weeks after the final AOM treatment (N = 15). Body weights were recorded weekly for 9 weeks after switching the diets and after that every two weeks until the end of the study. Colons were dissected, splayed open, and fixed for 16 hours in formalin. After briefly staining colons in methylene blue, ACF and adenomas were scored under a stereoscope.

**Table 1:** Nutrient compositions in regular chow and high fat diet.

Composition	Regular Chow (Pico Lab 5053)	High Fat Diet (Open Source Diet D12451)
Protein, kcal%	25	20
Fat, kcal%	13	45
Carbohydrate, kcal%	62	35
Kcal/gm	4.07	4.73

### Ethics statement

The study protocol was approved by the Chancellor's Animal Research Committee at the University of California, Los Angeles (ARC# 08–127). The study was carried out according to the recommendations in the Guide for the Care and Use of Laboratory Animals of the National Institutes of Health. Animals were humanely euthanized with CO_2_ inhalation followed by cervical dislocation.

### Body fat content

Prior to euthanasia, body fat and lean body mass were measured using an EchoMRI Whole Body Composition Analyzer.

### Quantitative PCR

Colons of mice sacrificed one day after the final dose of AOM were dissected and pieces from the mid colon were snap frozen in liquid nitrogen. RNA was prepared by a two-step method using Trizol (Invitrogen, Carlsbad, CA) and RNeasy mini columns (Qiagen, Valencia, CA). First strand cDNA was synthesized using SuperScript III First-Strand Synthesis kit primed with random hexamers, and gene expression was evaluated using TaqMan chemistry, Applied Biosystems 7500 Fast Real-Time PCR machine, and the following primers: TNF-α (Mm00443260_g1), IFN-γ (Mm01168134_m1), IL-6 (Mm00446190_m1), and IL-1β (Mm00434228_m1) (AB, Foster City, CA).

### Immunohistochemistry and apoptosis scoring

Colons from mice sacrificed one day after the final dose of AOM were fixed in formalin for 16 hours, and embedded in paraffin. Cross sections from at least 3 different pieces form the mid colon were H&E stained. Apoptosis was scored in the bottom half of the crypt by morphological features of nuclear condensation, nuclear fragmentation, and cytoplasmic blebbing. Proliferation was evaluated by Ki67 staining using Vectastain ABC kit and Vector DAB reagent according to manufacturers instructions (Vector Laboratories, Burlingame, CA). Lengths of the Ki67-positive proliferative zones were measured using Zeiss AxioVision software. For both apoptosis and proliferative zone lengths, at least 100 crypts were scored in total from three sections.

### Statistical analysis

Pair-wise comparisons of tumor multiplicity, proliferative zone lengths, and apoptosis were statistically compared using the non-parametric Mann-Whitney test, and pair-wise comparison of tumor incidence using Fisher's exact test. Real-time PCR analysis of gene expression was tested using two-way ANOVA. All analyses were performed using GraphPad Prism software (San Diego, CA).

## Results

### Diet-induced increase in body weight and body fat content promotes tumor development

**Figure 1:**
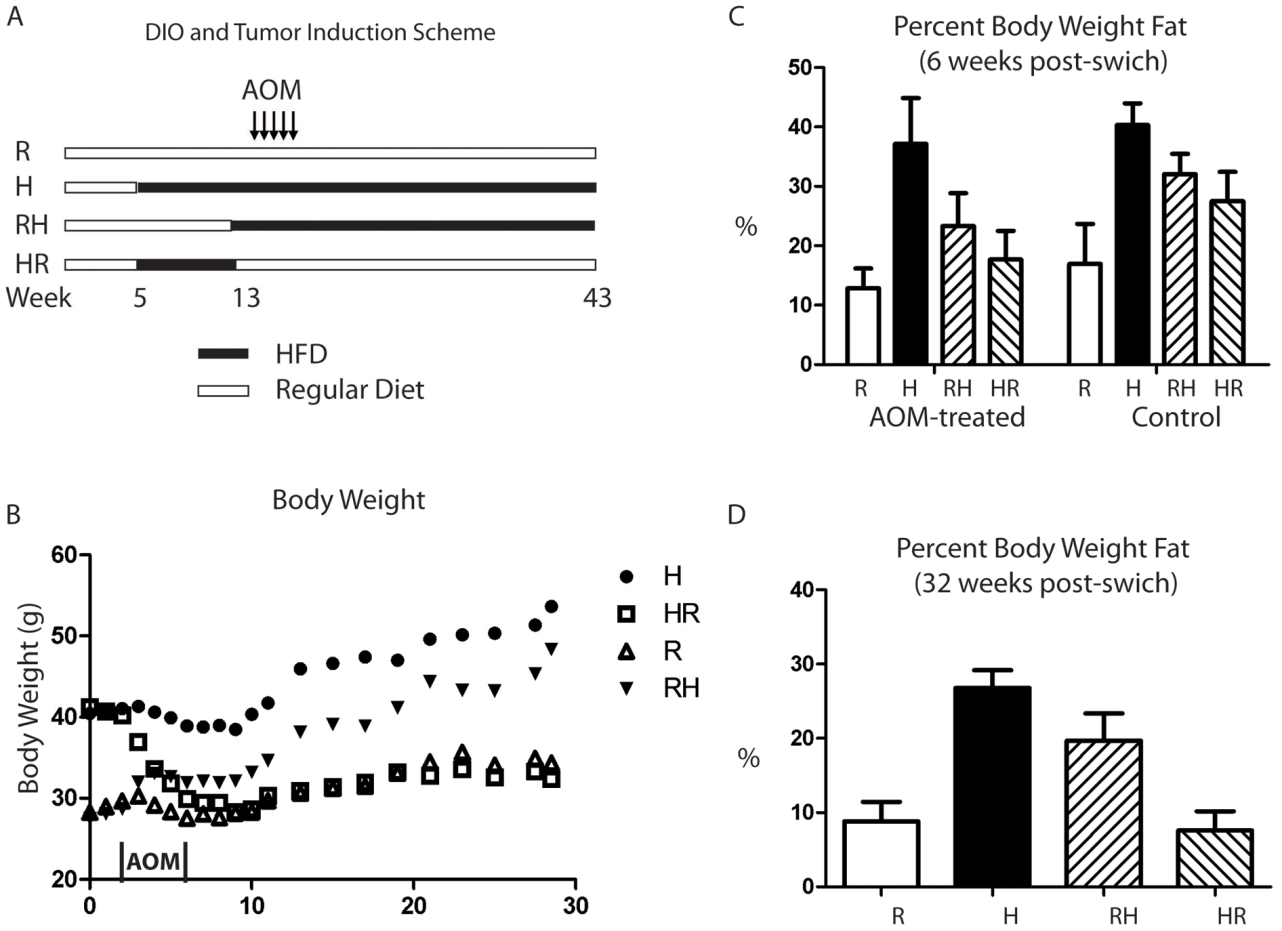
Body weights and fat content change following dietary switches. Schematic representation of diet induced obesity (DIO) and tumor induction with the age of the animal (A). Average body weight is shown for all groups of mice with time 0 reflecting the time when diets were switched in the *RH* and *HR* groups as described in methods. The period of 5 weekly AOM treatments is shown on the x-axis (B). Percent of body weight constituted by fat was measured by Echo MRI for animals euthanized 1 day after the last AOM treatment (5 animals per group) and age matched untreated animals that received the same dietary regimen (2 or 3 animals per group) (C). The same measurements were made for AOM treated animals 23 weeks after the final AOM-treatment (8 animals per group) (D).

To determine the effect of obesity, without the confounding effect of ongoing HFD, on colon tumor development, we designed four experimental feeding/carcinogen treatment groups (Fig. 1A). During the entire carcinogenic period (AOM treatment and after) *HR* and *R* group mice were both on the same regular diet, and *H* and *RH* group mice were both on HFD. After 8 weeks on either HFD or regular diet, mice in the *HR* group had comparable weights with those in the *H* group (Fig 1B). Similarly, mice in the *RH* had comparable weights with those in the *R* group. Mice in the *HR* group lost the most weight (25%) during the AOM treatment and reached the same weight as mice in the *R* group by 9 weeks after switching the diets. Mice in the *RH* group gained weight during and after AOM treatment but weighed less than those in the *H* group throughout the study (Fig. 1B). Although mice in the *HR* group had only slightly higher weight than mice in the *R* group one day after AOM treatment, their body fat content was 1.38-fold higher (Fig. 1C) as determined by Echo MRI. *RH* and *H* group mice had 1.81-fold and 2.89-fold higher body fat percentages, respectively, than *R* group mice after the AOM treatment (Fig. 1C), and at the end of the study they had 2.23-fold and 3.05-fold higher body fat contents, respectively, than the *R* mice (Fig. 1D).

**Figure 2:**
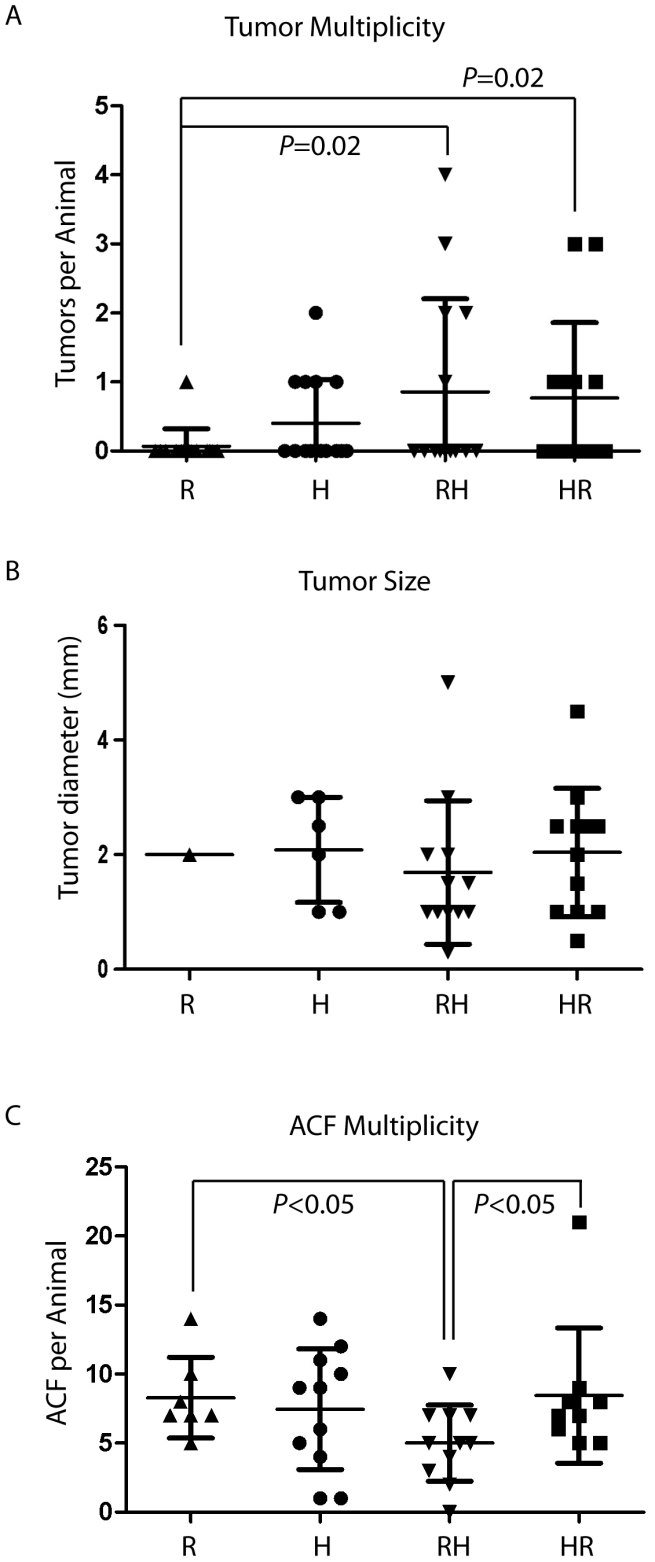
Obesity and HFD enhance colon tumor development. Colonic tumors were scored 23 weeks after the final AOM treatment and numbers observed by stereomicroscopy are shown (A). Tumor sizes were measured with a calibrated ocular reticle and are shown by group in (B). ACF per animal is shown in (C).

The great majority of the mice on a regular diet (group *R*) developed no adenomas with only 1/15 (0.07) animals developing a single adenoma (Fig. 2A), in accordance with previous studies showing that the C57BL6/J strain is relatively resistant to AOM-induced carcinogenesis [19]. Tumor incidence was increased in all groups that received HFD: 5/15 (0.33) in *H*, 5/14 (0.36) in *RH*, and 6/13 (0.46) in *HR*; with a significant increase in *HR* versus *R* (*P*<0.03, two-sided Fisher's exact test). Tumor multiplicity was also increased in all groups that received HFD (*H*, *HR*, and *RH*) relative to control animals (*R*), and a significant 10-fold increase in the mean tumor multiplicity was detected in *RH* and *HR* mice (0.9 in *RH* and 0.7 in *HR* vs 0.07 in *R*, *P* = 0.02 for both pairwise comparisons). No differences were observed in tumor size between the groups (Fig. 2B). The numbers of precancerous ACF were also scored. Interestingly, the only group that differed statistically was the *RH* group which had a small reduction in average ACF per animal (*P*<0.05, in pairwise comparisons with *HR* and *R*) (Fig. 2C).

### AOM-induced crypt apoptosis and proliferation are increased in mice switched from high fat to regular diet

**Figure 3:**
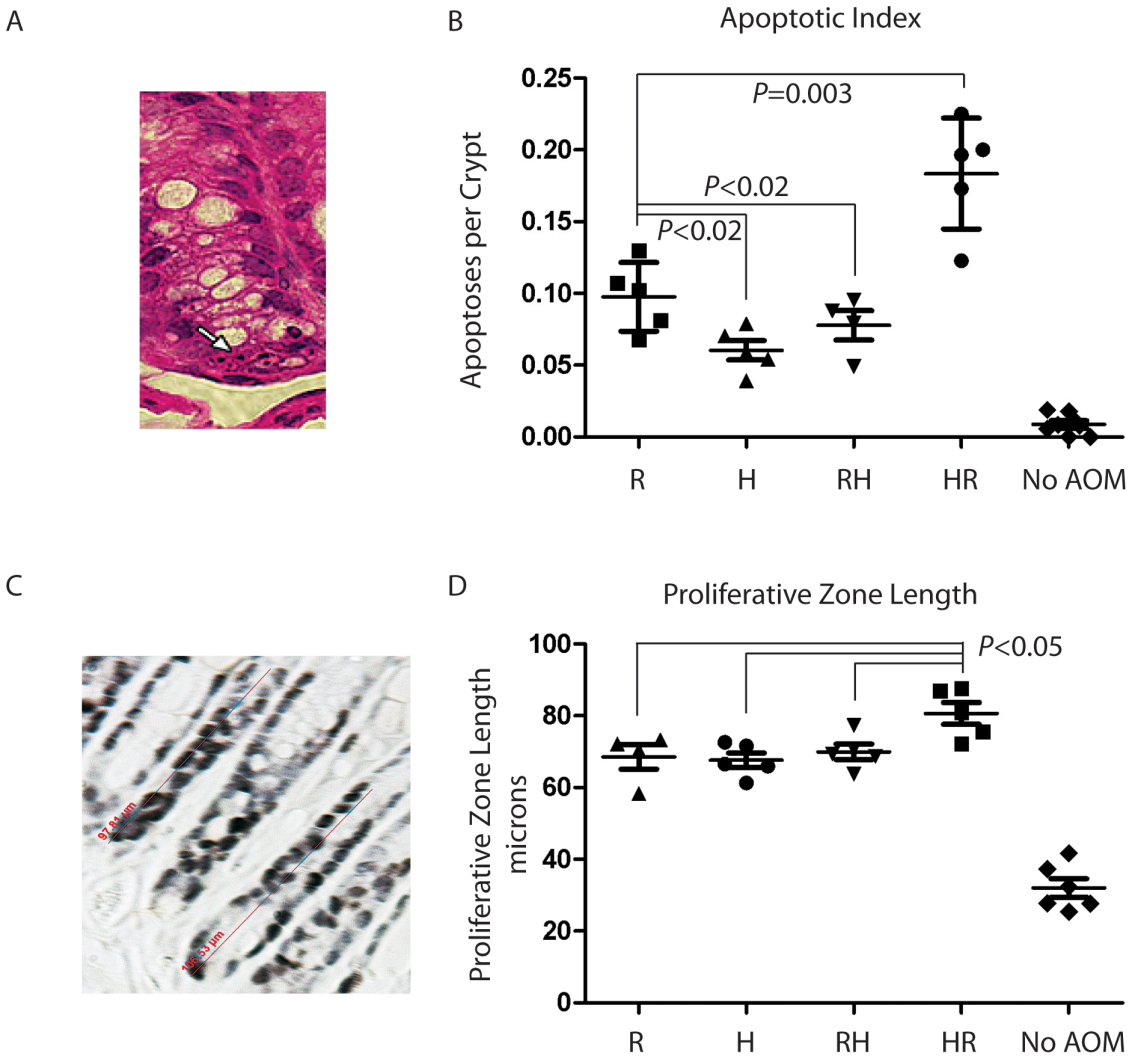
Obesity alters AOM induced proliferation and apoptosis. Animals were sacrificed 24 hrs after the final AOM treatment and apoptosis was scored in H&E stained sections by the following histological characteristics: cytoplasmic blebbing, nuclear condensation, and nuclear fragmentation. A representative apoptotic body is indicated by a white arrow in (A). Number and standard deviation of apoptoses per crypt is shown for each animal (B). Length of the proliferative zone was determined by Ki67 staining. A representative crypt with a measuring bar from AxioPhot software is shown in (C). Average length of the proliferative zone per animal is shown in (D).

*HR* group mice were significantly more sensitive to tumorigenesis than *R* group mice, yet their body weights and degree of adiposity mainly differed in the early stages after switching the diets. Because this period coincided with AOM treatment, we examined whether there was an interaction between obesity and cellular responses to AOM. AOM and other SN1 alkylating agents induce apoptosis in the colon, and AOM carcinogenicity is associated with increased proliferation in the colon during treatment [20]. In order to determine whether HFD altered AOM-induced crypt proliferation and apoptosis, 5 mice were euthanized one day after the last AOM injection. Apoptosis was quantified in half-crypts for nuclear condensation, fragmentation, and cytoplasmic blebbing (Fig. 3A). Without AOM, few apoptotic cells were detected in the crypts (Fig. 3B). AOM treatment significantly increased apoptosis in all groups relative to non-treated mice (Fig. 3B). Mice in the *HR* group showed the highest increase in apoptosis, and a significant increase was observed in the *HR* mice relative to the *R* mice (*P* = 0.003). Moreover, animals that received HFD during the carcinogenic period (groups *H* and *RH*) had significantly lower apoptotic indices relative to *R* group mice (*P*<0.02 for both pair-wise comparisons). We cannot rule out that this modest reduction at least partially resulted from the lower per body weight dose of AOM that these mice received. A similar trend was seen in proliferation, measured as Ki67-positive proliferative zone in colonic crypts (Fig. 3C). Lengthening of the proliferative zone was seen in all AOM-treated groups, again with the *HR* mice having the longest proliferative zone (*P*<0.05 for group *HR* in all pair-wise comparisons) (Fig. 3D). Taken together, *HR* mice appeared to have a greater cytotoxic response to AOM relative to all other groups.

### AOM treatment induces IFN-γ expression in the colon irrespective of diet

**Figure 4:**
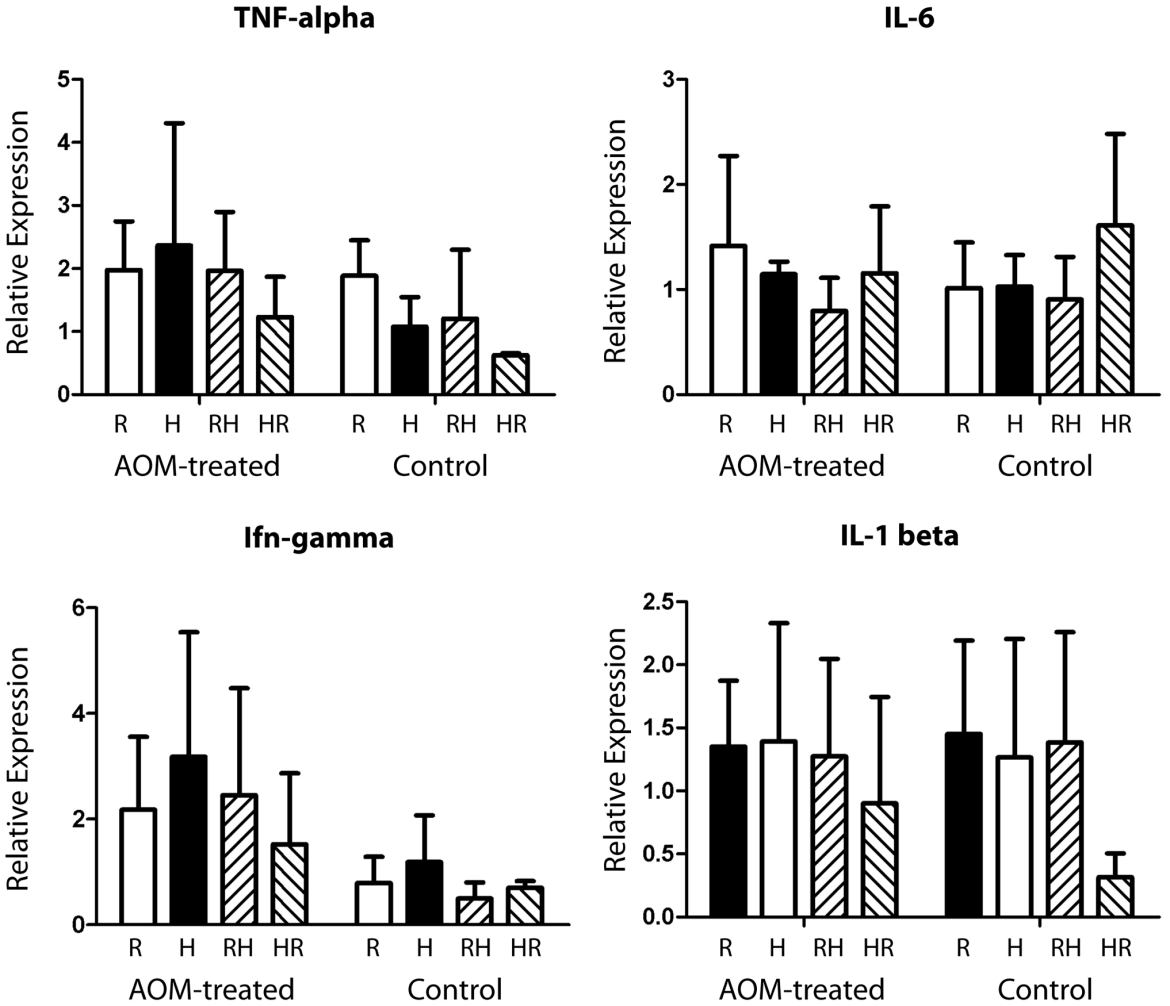
Obesity did not affect colonic cytokine expression. Gene expression of 4 cytokines, TNF-α, IL-6, IFN-γ, and IL-1β, was measured in RNA isolated from total colon 24 hours after the final AOM treatment (5 animals per group) and age matched untreated animals that received the same dietary regimen (2 or 3 animals per group). Relative expression values (mean +/− st. dev) are shown.

Inflammation is a potent tumor promoter, and both obesity and AOM-treatment have been linked with aberrant expression of pro-inflammatory cytokines [9], [21], [22]. For these reasons, we examined whether pro-inflammatory cytokine expression differed among our experimental groups with and without AOM treatment. Since the body weight and adiposity between *HR* and *R* groups were markedly different only before and during the AOM treatment, any alterations in cytokine expression derived from DIO would have been more pronounced at this time point than in the end of the study. Expression of TNF-α, IFN-γ, IL-6, and IL-1β was measured from mid-colon-derived RNA from AOM-treated and non-treated mice euthanized one day after the last AOM injection (N = 5). These four cytokines were chosen based on their well-established involvement in various inflammatory responses, as well as on previous findings in mouse models of AOM-induced carcinogenesis and obesity [21]–[24]. None of the HFD regimens (*H*, *HR*, *RH*) induced significant changes in the expression of these cytokines, when compared to control mice in the *R* group (Fig. 4). AOM treatment, however, induced a significant increase in IFN-γ expression in all four dietary groups, compared with non-treated mice (*P* = 0.02, Fig. 4). Thus, the differences in tumor sensitivity with different states of obesity do not appear to involve differences in expression of the pro-inflammatory cytokines tested.

## Discussion

The aim of this study was to determine whether obesity increases colon tumor development independent of the potential carcinogenic effects of ongoing intake of high dietary fat. There is a strong epidemiologic link between obesity and colonic neoplasia [2], [3]. The relative importance of chronic exposure to dietary fat versus the excess caloric intake leading to adipose tissue expansion is not completely understood as both can cause metabolic changes that favor tumor development. Here, we showed that obese mice switched from HFD to regular diet before carcinogen treatment developed more adenomas than those on a regular diet throughout, suggesting that factors associated with obesity - independently of maintained HFD and obesity - promote tumor development. Although our feeding model separated HFD and AOM exposures, we cannot completely rule out the potential long-term effects of the diet on the intestinal mucosa, such as an alteration in the colonic microbiota [25]. Nevertheless, the observed increase in tumor multiplicity in the obese (*HR*) mice was striking, considering that their body weight decreased rapidly during the carcinogenic period and reached that of the control mice by 9 weeks after switching the diets. This result parallels the epidemiological observation that adolescent obesity is associated with increased colon cancer incidence later in life [26], [27].

The period during which *HR* and *R* group mice differed significantly in adiposity coincided with AOM treatment. AOM is the most widely used DNA damaging agent in rodents that models human sporadic colon cancer initiation. This alkylating agent induces *O* ^6^-methylguanine formation in DNA, which can lead to G∶C to A∶T transition mutations, induction of apoptotic death and compensatory increases in proliferation. Both apoptosis and proliferation were significantly increased in the obese mice that were switched from high fat to regular diet relative to all other groups. Therefore the mechanism by which obese mice switched from HFD to regular diet (group *HR*) were more sensitive to carcinogenesis may differ from those in the mice that received HFD for the majority of the study (groups *RH* and *H*). Moreover, because all the obese groups (*H*, *RH*, and *HR*) showed increased tumor formation, but only *HR* mice showed enhanced apoptosis and proliferation, it seems that obesity did not affect the metabolism of this carcinogen. This is further supported by the observation that *HR* and *R* group mice developed identical numbers of ACF and hence AOM unlikely affected the early fixation of mutations in the obese mice. On the other hand, since the same dose of AOM was given to all animals irrespective of weight, the smaller per kilogram dose in the *H* and *RH* groups may have diminished the effective amount of carcinogen in the heavier groups.

Colon cancer typically develops from a hyperproliferative epithelial crypt into a cluster of such abnormal crypts known as ACF, which can then grow into a benign, localized adenoma, and subsequently into an invasive carcinoma that eventually metastasizes to distant organs [28], [29]. We observed an increase in adenomas, but not in ACFs, with HFD. In fact, *RH* group was the only one with a modest but significant reduction in the number of ACFs, while these mice had the highest mean tumor number. These results suggest that obesity, without ongoing HFD, enhances colon tumor formation without enhancing ACF formation or tumor growth rates. This finding differs from previous reports on increased ACF multiplicity in genetic or DIO models of obesity combined with chemical carcinogenesis [7], [16], [30], [31]. These differences could reflect the timing at which ACF were quantified. Alternatively, Hata *et al*. found an increase in β-catenin accumulated crypts (BCACs), but not ACFs, in a monosodium glutamate-induced mouse model of diabetes and obesity [32]. BCACs are dysplastic, rather than hyperplastic, mucosal lesions identified by nuclear accumulation of beta-catenin. These investigators reported that the insulin like growth factor receptor 1 (IGF-1R), a candidate mediator of the tumor-promoting effect of obesity, is expressed specifically in BCACs [32]. It is hence possible that BCACs represent a more accurate premalignant lesion for obesity/hyperinsulinemia-related tumorigenesis.

We also showed increases in tumor multiplicity in animals that were fed HFD throughout the carcinogenesis period relative to regular diet fed animals. In general our results are in concordance with previous studies using DIO and genetic models [7], [14], [18]. Studies in murine genetic models have attempted to dissect the contributions of these different factors and have provided important insights into our mechanistic understanding of obesity-associated colon cancer. Mice with the KK mutation and the agouti gene (*Ay*) are diabetic and severely obese, displaying hyperphagia, polydypsia, glucose intolerance, hyperlipidemia, and hyperinsulinemia [33]. These mice are extraordinarily sensitive to AOM-induced ACF, adenomas, and adenocarcinomas [30]. Other genetic models of obesity and diabetes, including the leptin- or leptin receptor-deficient obese mice (ob/ob or db/db), are also more susceptible to alkylation-induced ACF [7], [18]. Evidence that hyperinsulinemia promotes tumor development comes from a fatless mouse (A-ZIP). Despite having no mature adipose tissue these mice develop hyperinsulinemia, insulin resistance, and elevated systemic levels of pro-inflammatory cytokines, triglycerides, and free fatty acids. Importantly, they show a marked increase in sensitivity to skin and mammary carcinogenesis [34].

While we did not observe colonic expression differences in inflammatory cytokines at the mRNA level, it is possible that various adipokines, hormones, and pro-inflammatory cytokines are potential mediators of the effect of obesity on colon cancer development. Mouse studies using DIO have shown increased colonic TNF-α expression in HFD-fed animals [22], [35]. Liu et al. reported that HFD for 12 weeks, without carcinogen treatment, increased colonic TNF-α expression by 72% [22]. Ding et al. reported increased ileal, rather than colonic, TNF-α expression following HFD feeding, which preceded hyperinsulinemia and weight gain [23]. IL-6 has been shown to be upregulated in the distal colon after HFD feeding [16]. In a study by Mentor-Marcel et al. (2009), various pro-inflammatory cytokines, including IL-6 and IFN-γ were increased in the serum of ob/ob mice in the pre-malignant but not advanced stages of AOM-induced colon tumor development [24]. Furthermore, the levels of IL-6 were reduced in these animals upon dietary intervention, in both serum and colonic mucosa [24]. In addition to pro-inflammatory cytokines, elevated levels of insulin, which can stimulate pro-survival and growth signaling in epithelial cells, is a key candidate for mediating the effect of obesity on colon cancer development [10].

In summary, all mice that were fed HFD showed an increase in the number of adenomas, in particular those switched from HFD to regular diet before carcinogen exposure. This study hence demonstrates that prior HFD-induced obesity, even without maintained HFD and weight gain, promotes colon tumor development. Further studies are needed to determine the respective roles of various adipokines, hormones, and cytokines in this complex process.
